# Thermoelectric SnS and SnS-SnSe solid solutions prepared by mechanical alloying and spark plasma sintering: Anisotropic thermoelectric properties

**DOI:** 10.1038/srep43262

**Published:** 2017-02-27

**Authors:** Asfandiyar, Tian-Ran Wei, Zhiliang Li, Fu-Hua Sun, Yu Pan, Chao-Feng Wu, Muhammad Umer Farooq, Huaichao Tang, Fu Li, Bo Li, Jing-Feng Li

**Affiliations:** 1 State Key Laboratory of New Ceramics and Fine Processing, School of Material Science and Engineering, Tsinghua University, Beijing 100084, China; 2 Xinjiang Inspection Institute of Special Equipment, Urumqi, 830011, China; 3 Advanced Materials Institute, Graduate School at Shenzhen, Tsinghua University, Shenzhen, 518055, China; 4 School of Physics and Energy, and Shenzhen Key Laboratory of Sensor Technology, Shenzhen University, Shenzhen, 518060, China

## Abstract

*P*–type SnS compound and SnS_1−x_Se_x_ solid solutions were prepared by mechanical alloying followed by spark plasma sintering (SPS) and their thermoelectric properties were then studied in different compositions (x = 0.0, 0.2, 0.5, 0.8) along the directions parallel (//) and perpendicular (⊥) to the SPS–pressurizing direction in the temperature range 323–823 Κ. SnS compound and SnS_1−x_Se_x_ solid solutions exhibited anisotropic thermoelectric performance and showed higher power factor and thermal conductivity along the direction ⊥ than the // one. The thermal conductivity decreased with increasing contents of Se and fell to 0.36 W m^−1^ K^−1^ at 823 K for the composition SnS_0.5_Se_0.5_. With increasing selenium content (x) the formation of solid solutions substantially improved the electrical conductivity due to the increased carrier concentration. Hence, the optimized power factor and reduced thermal conductivity resulted in a maximum *ZT* value of 0.64 at 823 K for SnS_0.2_Se_0.8_ along the parallel direction.

Thermoelectric (TE) devices have the ability to convert waste heat directly into electrical energy and vice versa, which have advantages of no moving parts, no emission of any greenhouse gases, quiet operation, being free from liquid fuels and high reliability^1^. The conversion efficiency of thermoelectric devices can be characterized by the dimensionless figure of merit, *ZT* = *S*^*2*^*σT*/*κ* where the parameters *S* is the Seebeck coefficient, *σ* is the electrical conductivity, *κ* is the thermal conductivity which consists of the lattice conductivity (*κ*_L_) and the electronic thermal conductivity (*κ*_e_) and *T* is the absolute temperature^2^.

SnS and SnSe have received increasing attention as new thermoelectric materials with abundant resources and better environmental compatibility^3^,^4^. These materials in their pristine form possess high Seebeck coefficient and low thermal conductivity but low electrical conductivity due to the low carrier concentration which results in low *ZT* values. Electrical conductivity can be enhanced by optimizing carrier concentration through elemental doping and band convergence^5^,^6^,^7^,^8^,^9^,^10^, while thermal conductivity can be suppressed *via* forming solid solutions (alloying)^11^,^12^, nanostructure architecture and microstructure modulation^13^,^14^,^15^,^16^,^17^,^18^,^19^ by virtue of intensifying phonon scattering at atomic, nano and micro scales, respectively. Among these strategies, forming solid solutions^20^,^21^,^22^,^23^ has also been revealed as an effective way to modify the band structures: it can alter the band shape^24^ (effective mass), change the band gap^25^ (related to bipolar effect), and also affect the relative position of different bands^10^,^26^ (band alignment and convergence), thus directly determining charge transport and thermoelectric performance. Recently a *ZT* value up to 0.6 at 873 K was achieved in Ag–doped SnS polycrystals by Tan *et al*.^4^, which is 275% higher than the undoped samples^11^,^27^. SnSe is the heavier analogue of SnS, and both crystalize in highly anisotropic layered orthorhombic structure (*Pnma*) at room temperature and convert to *Cmcm* at high temperatures^3^,^9^,^11^. Optical properties on the two materials has been studied extensively in the past^28^,^29^, and recent attention has been paid to their thermoelectric properties^3^,^4^,^9^,^13^,^19^. Guo *et al*.^30^ comparatively calculated the *ZT* values of SnSe and SnS using first principle and the Boltzmann transport, and found that SnSe has larger optimal *ZT* than SnS. Recently an unprecedented *ZT* of 2.6 at 973 K was observed in SnSe single crystal along *b*–axis due to moderate power factor and ultra–low lattice thermal conductivity^3^. Nevertheless, due to the rigorous and low–efficiency of synthetic process of the single crystals, most of the thermoelectric materials are expected to work in polycrystalline forms, which can be synthesized through low cost powder metallurgical processes. A typical example is a combination of mechanical alloying (MA) and spark plasma sintering (SPS). The advantage of MA is to get fine powders in a short alloying time and low fabrication cost while SPS treatment can achieve high density close to the theoretical value and keeps the original microstructure using faster rate which also help to avoid the coarsening of grains.

In this work SnS_1−x_Se_x_ solid solutions with different x values were obtained by MA combined with SPS. Although relatively low thermal conductivity was achieved in the composition SnS_0.5_Se_0.5_ rather than SnS_0.2_Se_0.8_ that was reported previously^11^, the power factor (*PF* = *S*^*2*^*σ*) was greatly optimized in the composition SnS_0.2_Se_0.8_ than in the composition SnS_0.5_Se_0.5_. Hence, the optimized power factor along with reduce thermal conductivity resulted in a high *ZT* value of 0.64 at 823 K for SnS_0.2_Se_0.8_ without doping along the direction parallel to the SPS–pressurizing direction.

## Results and Discussion

### Phase and microstructure

**Figure 1:**
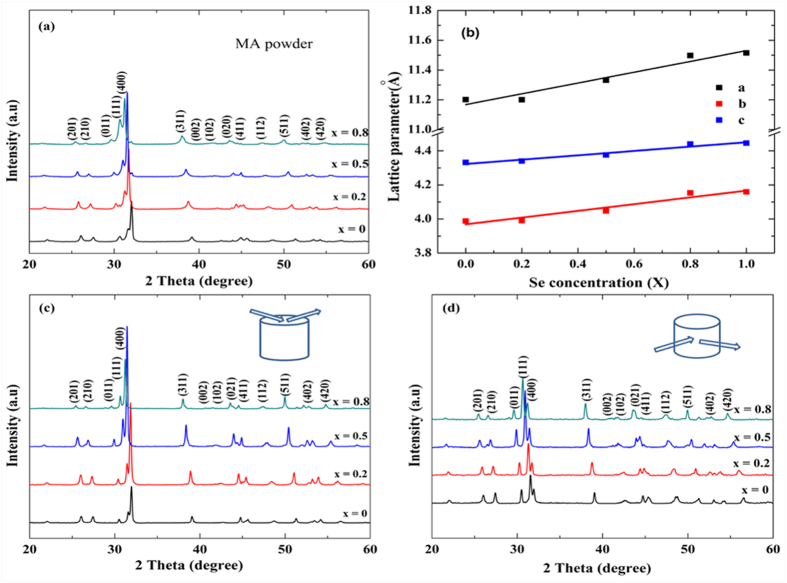
(**a**) XRD patterns of SnS_1−x_Se_x_ (x = 0, 0.2, 0.5, 0.8) MA–powders. (**b**) Lattice parameters of SnS_1−x_Se_x_ solid solutions varying with the increase in Se content (x). (**c,d**) XRD patterns of SnS_1−x_Se_x_ bulk specimens cut along two different directions.

**Figure 2:**
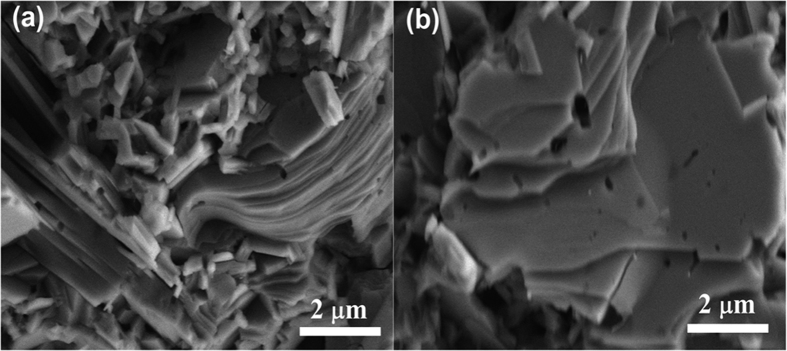
FESEM morphology of fractured surfaces of SnS bulk sample (**a**) parallel (//) and (**b**) perpendicular (⊥) to the SPS–pressurizing direction.

SnS and SnSe compounds are crystallized in a layered structure with orthorhombic *Pnma* space group (PDF#39–0354 and PDF#48–1224) at room temperature and show a phase transition from *Pnma* to the *Cmcm* symmetry at high temperatures (858 K for SnS and 803 K for SnSe)^3^,^4^,^5^. Figure 1 represents the XRD patterns of the pure SnS and SnS_1−x_Se_x_ solid–solution powders and bulk samples along two different directions. The result confirms that single–phase SnS_1−x_Se_x_ solid solutions with an orthorhombic crystal structure were formed. A shift in *2θ* of all peaks toward the lower angle was observed with increasing x. The lattice parameters of SnS_1−x_Se_x_ solid solution powders expand linearly with increase in Se contents (x), which are in accordance with the Vegard’s law (Fig. 1b), indicating that the smaller S atoms were replaced by the larger Se. Obvious anisotropy is seen from the XRD patterns of the bulk specimens cut along the two directions. For the specimen cut perpendicular to the SPS pressure (specimen_⊥_, Fig. 1c), (400) peak is much stronger than the specimen_//_ (Fig. 1d). In fact, the calculated orientation factors of (400) are 0.3 and 0.13 for specimen_⊥_ and specimen_//_, respectively. This anisotropy has been widely observed and explained in previous studies on polycrystalline SnS, SnSe and other layered compounds^3^,^11^. From the FESEM images we can see an obvious thin–platelet morphology (Fig. 2a,b). The cross-section_⊥_ shows a flat surface (parallel to the (400) crystallographic plane) of platelets compare to other directions, which is in good agreement with the anisotropic XRD patterns shown in Fig. 1c and d. Inside the grains pores are present with relative densities ranging from 96.3% to 91.2%. This phenomenon is possibly related to the slight volatilization of S and Se that is also seen from the composition detected by ICP shown in Table 1.

### Electrical transport

**Figure 3:**
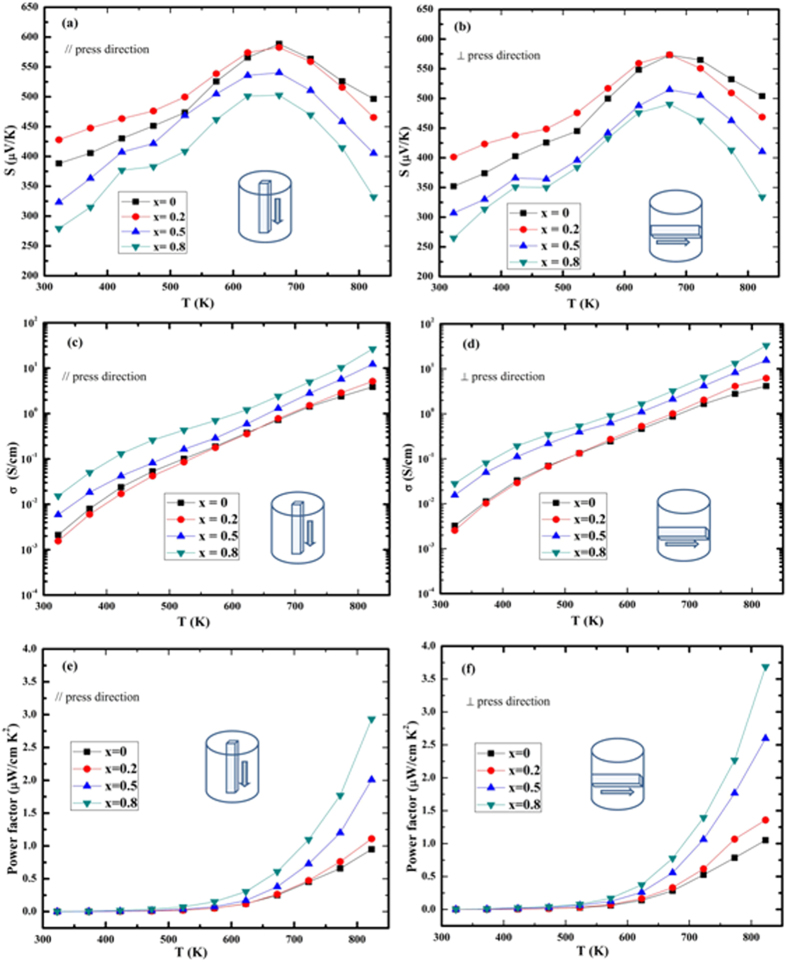
Temperature dependence of the (**a,b**) Seebeck coefficient, (**c,d**) electrical conductivity and (**e,f**) power factor of the SnS_1−x_Se_x_ (x = 0, 0.2, 0.5, 0.8) solid solutions (**a,c,e**) along and (**b,d,f**) perpendicular to the SPS-pressurizing direction.

The Seebeck coefficient (*S*) of SnS_1−x_Se_x_ solid solutions as a function of temperature along two directions is shown in Fig. 3. *S*_⊥_ is 352 μV/K at 323 K, which is well consistent with our previous reports^4^,^27^ but somewhat lower than the data reported by Han *et al*.^11^ In fact, over the whole temperature range, *S*_*//*_, *S*_⊥_ value of SnS (this work) and *S*_⊥_ in our previous work^4^,^27^ are lower than the published data^11^. The difference in the *S* values probably originates from the synthesis processes of the samples (mechanical alloying, SPS and melting, SPS). Similar difference also exists in the *S* value of SnSe prepared by different methods^10^,^31^. *S* decreases with increase in Se contents (x) due to the increased carrier concentration as well as slightly enhanced mobility (as shown in Table 2). *S* along both directions of all the samples increases in the temperature range 323–673 K and then turns to decrease with increasing temperature due to bipolar conduction. It is also seen that *S*_⊥_ is lower than *S*_//_ for all the compositions over the whole temperature range.

Figure 3(c) and (d) shows the electrical conductivity (*σ*) of SnS_1−x_Se_x_ solid solutions in the temperature range 323–823 K in the two directions. The *σ* of the SnS_1−x_Se_x_ solid solutions increases with rising temperature. *σ*_⊥_ of SnS is 3.24 × 10^−3^ S/cm at 323 K, which is comparable to our previous studies^4^,^27^ but greatly lower than Han’s work^11^. For solid solutions, *σ* is also lower than the same composition in ref. 11. This phenomenon is ascribed to the lower carrier concentration (*n*_H_) and mobility (*μ*_H_) in this work as shown in Table 2. The lower mobility can be intuitively understood since samples here prepared by MA should contain abundant defects that act as scattering centers of carriers. The reason for the lower carrier concentration is not quite clear. We tentatively argue that the off stoichiometry, probably the existence of S deficiencies, may be important. Recalling the Seebeck coefficient as mentioned above, it is also smaller in this study, which seems to be in contrary to the common sense that a lower *n*_H_ usually gives a larger *S*. However, this simple prediction is reliable only when the dominant scattering mechanisms are similar, which need further investigation. *σ* decreases with increase of Se content (x) for x = 0.2 at 323 K, which is due to the impaired carrier mobility by alloying. With even more Se, *σ* increases with x, which is mainly due to increased *n*_H_. It is also seen that over the whole temperature range all the SnS_1−x_Se_x_ solid solutions, *σ*_//_ is lower than *σ*_⊥_ one due to the preferred orientation of the (400) plane in this direction, which is related to the larger effective mass and the consequentially lower carrier mobility along this direction^3^. The highest electrical conductivities of 33.1 S/cm and 27 S/cm are obtained at 823 K for the composition SnS_0.2_Se_0.8_ along the directions ⊥ and // to the SPS–pressurizing direction, respectively. Figure 3(e) and (f) present the power factors (*PF* = *S*^*2*^*σ*) of all the SnS_1−x_Se_x_ samples versus selenium content (x). Over the entire temperature range, the *PF*_⊥_ is higher than *PF*_//_. The maximum *PF*_⊥_ and *PF*_//_ of the composition SnS_0.2_Se_0.8_ at 823 K are 3.7 μW cm^−1^ K^−2^ and 2.93 μW cm^−1^ K^−2^, respectively.

### Thermal transport

**Figure 4:**
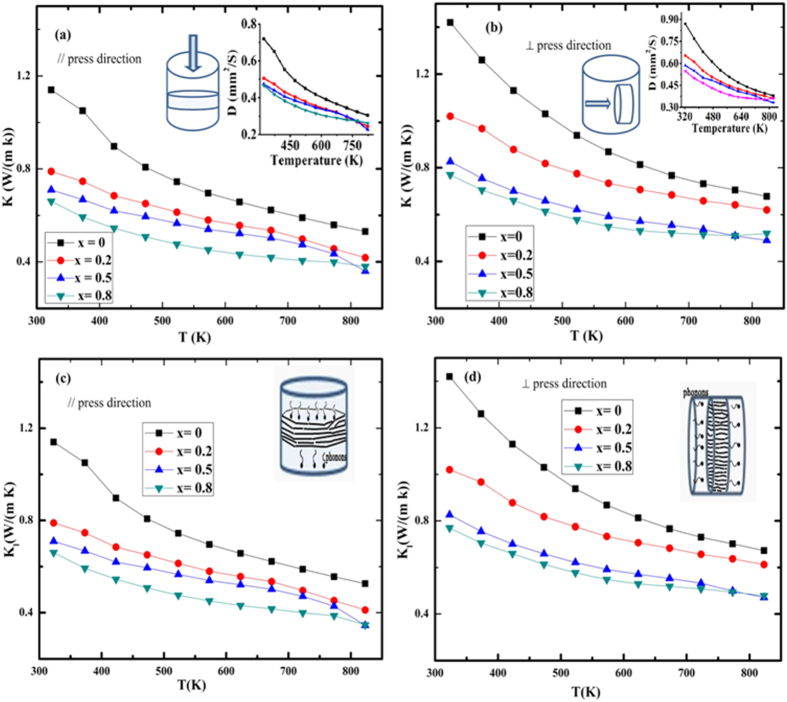
Temperature dependence of the total thermal conductivity (the inset shows the thermal diffusivity) of the SnS_1−x_Se_x_ (x = 0, 0.2, 0.5, 0.8) solid solutions (**a**) parallel (//) and (**b**) perpendicular (⊥) to the SPS–pressurizing direction. The lattice thermal conductivity of the SnS_1−x_Se_x_ solid solution (**c**) parallel (//) and (**d**) perpendicular (⊥) to the SPS–pressurizing direction.

The temperature dependence of total thermal conductivity (*κ*) and lattice thermal conductivity (*κ*_L_) of the SnS_1−x_Se_x_ solid solutions is shown in Fig. 4. Inset of Fig. 4(a) and (b) represents the thermal diffusivity along the // and ⊥ directions. Over the whole temperature range, *κ*_*//*_ of all the samples are lower than the *κ*_⊥_. *κ* decreased with x and the lowest value 0.36 W m^−1^ K^−1^ at 823 K was obtained for SnS_0.5_Se_0.5_ along the //direction. For all the SnS_1−x_Se_x_ samples *κ* decreases with increasing temperature. For x = 0.5 *κ* decreases faster above 773 K than for x = 0.8, which leads to the lower *κ* in the SnS_0.5_Se_0.5_ composition. Similar behavior for this composition has also been found in Han’s work^11^, but the reason is still unclear. *κ* and *κ*_L_ of polycrystalline SnS compound along the direction ⊥ to the SPS–pressurizing direction are all 1.4 W m^−1^ K^−1^ which is consistent with the previously reported value for SnS compound measured along the same direction^4^. *κ*_L_ decreases with increase of selenium content (x) due to the alloying effect caused by different atomic masses of Se (79.86 g/mol) and S (32.07 g/mol) and strain field fluctuation caused by difference in atomic radii (1.91 Å of Se and 1.84 Å of S). *κ*_l_ decreases with increasing temperature for all the SnS_1−x_Se_x_ samples due to the intensified Umklapp process. Due to the anisotropy in microstructure, *κ*_//_ is considerably lower than *κ*_⊥_.

### Figure of merit (*ZT*)

**Figure 5:**
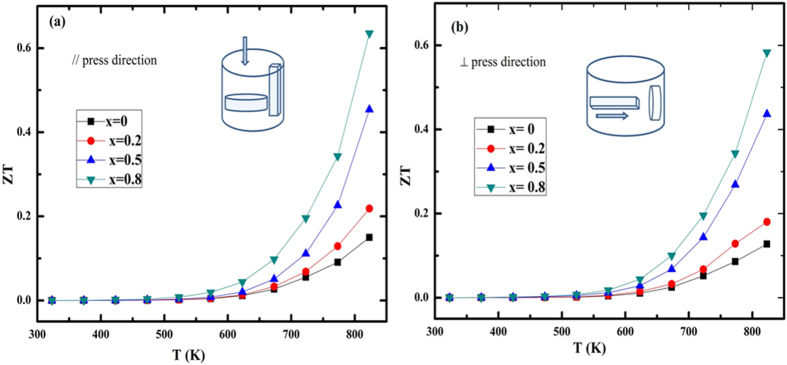
Temperature dependence of the *ZT* values of SnS_1−x_Se_x_ (x = 0, 0.2, 0.5, 0.8) solid solutions (**a**) parallel (//) and (**b**) perpendicular (⊥) to the SPS–pressurizing direction.

The *ZT* values of all the SnS_1−x_Se_x_ (x = 0, 0.2, 0.5, 0.8) solid solutions along the directions // and ⊥ to the SPS–pressurizing one were calculated from the combination of anisotropic electrical and thermal transport properties (Fig. 5). Over the whole temperature range, *ZT*_//_ values were higher than *ZT*_⊥_, which comes mainly from the greatly suppressed thermal conductivity although power factors are lower along this direction. *ZT*_*//*_ for SnS is 0.15 at 823 K and increases with Se content. The *ZT* values of all the SnS_1−x_Se_x_ samples increased rapidly with rising temperature and a maximum value of 0.64 at 823 K for composition SnS_0.2_Se_0.8_ was obtained along the direction // to the SPS–pressurizing direction.

## Conclusion

*P*–type SnS compound and SnS_1−x_Se_x_ solid solutions were successfully prepared by mechanical alloying combined with spark plasma sintering. High anisotropy among all the transport properties has been observed, *i.e*. higher *PF* and *σ* along the direction perpendicular to the SPS–pressurizing direction than the parallel one. Se substitution subsequently increased the hole carrier concentration from 9.02 × 10^14^ cm^−3^ in SnS to 2.10 × 10^17^ cm^−3^ in SnS_0.2_Se_0.8_, which results in increased *σ* and *PF*. A high *ZT* value of 0.64 has been obtained for the composition SnS_0.2_Se_0.8_ at 823 K along the direction // to the SPS–pressuring direction due to the reduced *κ* and optimized *PF*. SnS and all the SnS_1−x_Se_x_ solid solutions exhibit higher thermoelectric performance along the direction parallel to the SPS–pressurizing direction than the other one. Moreover, although a low *κ* of 0.36 at 823 K was observed in SnS_0.5_Se_0.5_, its maximum *ZT* value 0.45 is still lower than the SnS_0.2_Se_0.8_ owing to its low *σ* and *PF*. However, both compositions (SnS_0.5_Se_0.5_, SnS_0.2_Se_0.8_) are promising candidates for further thermoelectric investigations towards higher performance through proper *p–*type doping to increase *σ* and *PF*.

## Materials and Methods

### Materials

The experiments started from raw elements Sn (99.99% powder), S (99.99% shots) and Se (99.9% powder). The raw materials with a total mass of 20 g were weighed according to the nominal compositions of SnS_1−x_Se_x_ (x = 0, 0.2, 0.5, 0.8), loaded into a stainless steel jar of volume 250 ml with stainless steel balls of different diameters and masses (10 mm, ~4 g and 6 mm, ~1 g) in a dry argon–filled glow box and then subjected to MA. The numbers of large and small balls were 44 and 250, respectively. Ball milling was conducted continuously at 450 rpm for 15 hours. The MA–derived powders were then loaded into a graphite die with an inner diameter of 15 mm and was spark plasma sintered at 903 K in vacuum for 7 min under axial pressure of 50 MPa. Finally, a cylinder shaped samples of average thickness about 12 mm and 15 mm in diameter were obtained. The phase structure of all the samples were examined by X–ray diffraction (XRD) using Cu Kα radiation (λ = 1.5418 Å). The morphology of the bulk samples was observed through a field emission scanning electron microscope (FE–SEM, JSM–7001 JEOL, Japan). The chemical composition was analyzed by inductively coupled plasma optical emission spectroscopy (ICP-OES, VISTA-MPX, Varian, USA). To investigate the electrical and thermal transport properties along the directions parallel and perpendicular to the SPS–pressurizing direction, bar–shaped specimens (2 × 2 × 11 mm) and disks (φ6 × 1.2 mm) were cut from bulk samples and polished with fine–grit sandpaper. The simultaneous measurement of the Seebeck coefficient and electrical resistivity was done using bar–shaped samples by a Seebeck coefficient/electrical measuring system (ZEM–2, ULVAK–RIKO, Japan), under partial helium pressure in the temperature range 323–823 K. The Hall coefficients were obtained at room temperature by the Van der Paw technique under a reversible magnetic field of 0.52 T (8340 DC, Toyo Japan). The density (*d*) of the bulk samples were measured by the Archimedes method. The *C*_*P*_ values of SnS and SnSe were obtained from previous work^3^,^32^, and the values for solid solutions were calculated using linear average. The disk–shaped samples were used to measure the thermal diffusivity (*D*) by the laser flash model (TC–9000, ULVAC–RIKO Japan). The heat capacity and density of the SnS_1−x_Se_x_ samples can be found in Table S1 (Supplementary Information) and Table 1. Finally, the total thermal conductivity was calculated using *κ* = *DC*_*P*_*d*.

## Additional Information

**How to cite this article:** Asfandiyar *et al*. Thermoelectric SnS and SnS-SnSe solid solutions prepared by mechanical alloying and spark plasma sintering: Anisotropic thermoelectric properties *Sci. Rep.* **7**, 43262; doi: 10.1038/srep43262 (2017).

**Publisher's note:** Springer Nature remains neutral with regard to jurisdictional claims in published maps and institutional affiliations.

## Supplementary Material

Supplementary Information

## Figures and Tables

**Table 1:** Nominal composition, real composition and density of SnS_1−x_Se_x_ at room temperature.

Se content (x)	0	0.2	0.5	0.8
**Nominal comp %**	50:50	50:40:10	50:25:25	50:10:40
**Real comp %**	51.3:48.7	51.7:38.7:9.60	51.1:24.3:24.6	50.6:10.5:38.8
**Density (g/cm**^**3**^)	4.97	5.17	5.32	5.45

**Table 2:** Carrier concentration and mobility of SnS_1−x_Se_x_ measured on the specimens cut perpendicular to the SPS pressurizing direction (⊥, in-plane measurement) at room temperature and Seebeck coefficient and electrical conductivity along (//) and (⊥) directions obtained at 323 K.

Measured Parameters	Se concentration (x)			
	x = 0	x = 0.2	x = 0.5	x = 0.8
***n***_**H**_ **(⊥) (10**^**17**^** cm**^**-3**^)	0.00902	0.505	1.12	2.10
***μ***_**H**_ **(⊥) (cm**^**2**^**/Vs)**	7.32	0.523	0.438	0.645
***S***_**//**_ **(μV/K)**	388	428	323	279
***S***_**⊥**_ **(μV/K)**	352	401	307	265
***σ***_**//**_ **(S/m)**	0.211	0.155	0.588	1.53
***σ***_**⊥**_ **(S/m)**	0.324	0.255	1.55	2.80
