# Supplementary material for: Thermoelectric SnS and SnS-SnSe solid solutions prepared by mechanical alloying and spark plasma sintering: Anisotropic thermoelectric properties
Source: Sci Rep. 2017 Feb 27;7:43262. doi: 10.1038/srep43262 (PMC5327431; doi:10.1038/srep43262)
Supplement: Supplementary Information [file srep43262-s1.pdf]

Supporting Information:

Thermoelectric SnS and SnS-SnSe solid solutions prepared by mechanical alloying and spark plasma sintering: Anisotropic thermoelectric properties

Asfandiyar, [a] *Tian-Ran Wei,* [a] *Zhiliang Li,* [a]* *Fu-Hua Sun,* [a] *Yu Pan,* [a] *Chao-Feng Wu,* [a] *Muhammad Umer Farooq,* [b] *Huaichao Tang,* [a] *Fu Li,* [c,d] *Bo Li,* [c] *Jing-Feng Li* [a]*

[a]State Key Laboratory of New Ceramics and Fine Processing, School of Material Science and Engineering, Tsinghua University, Beijing100084, China.

[b]Xinjiang Inspection Institute of Special Equipment, Urumqi, 830011, China.

[c]Advanced Materials Institute, Graduate School at Shenzhen, Tsinghua University, Shenzhen, 518055, China

[d]School of Physics and Energy, and Shenzhen Key Laboratory of Sensor Technology, Shenzhen University, Shenzhen, 518060, China

Corresponding Author: [jingfeng@mail.tsinghua.edu.cn, 460407475@qq.com]

1. The heat capacity (C_P) of $SnS_{1-x}Se_x$ (x = 0.2, 0.5, 0.8).

The heat capacity values for $SnS_{1-x}Se_x$ are listed in Table S1, which were calculated by linear fitting from the Cp values of SnS and SnSe.

Figure S1 The heat capacity (C_P) values of the SnS and SnSe obtained from the previous studies[1,2].

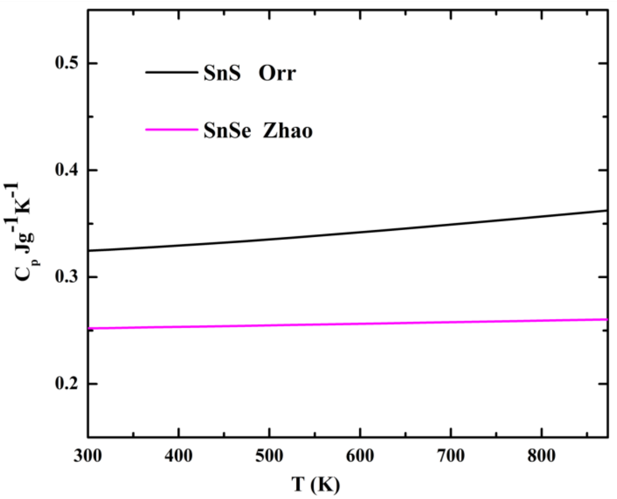

Table S1 The heat capacity (C_P) values of $SnS_{1-x}Se_x$ (x = 0, 0.2, 0.5, 0.8) calculated by linear fitting.

Se concentration (x)	x = 0	x = 0.2	x = 0.5	x = 0.8
Temperature (K)	Heat capacity ($Jg^{-1}K^{-1}$)			
323	0.32562	0.31096	0.28897	0.26697
373	0.32803	0.31303	0.29055	0.26806
423	0.3307	0.3153	0.2922	0.2691
473	0.33363	0.31779	0.29404	0.27029
523	0.33677	0.32046	0.29599	0.27152
573	0.3401	0.32327	0.29803	0.27279
623	0.34358	0.32618	0.30009	0.27399
673	0.34719	0.32925	0.30233	0.27442
723	0.35089	0.33234	0.3045	0.27557
773	0.35467	0.33551	0.30677	0.27603
823	0.35847	0.3387	0.30905	0.2759

2. The orientation factor

The orientation factor was calculated for the (111) and (400) planes by using the following equations[3].

$$F = \frac{P - P_0}{1 - P_0} \qquad (1)$$

$$P_0 = \frac{I_0(h00)}{\sum I_0(hkl)} \qquad (2)$$

$$P = \frac{I(h00)}{\sum I(hkl)} \qquad (3)$$

Where P and P_0 are the integrated intensities of all (*h00*) planes to the intensities of all (*hkl*) planes for preferentially and randomly oriented samples.

3. Additional experiments for the stability of electrical transport properties

To ensure the stability of transport properties at high temperature annealing were performed of one of the sample ($SnS_{0.2}Se_{0.8}$) at 773 K for four days. The electrical transport properties of the annealed sample along the two direction // and ⊥ were measured in the temperature range 323-823 K (Figure S2). For comparison, the data of SPS-ed $SnS_{0.2}Se_{0.8}$ (without annealing) are also given. No significant difference is seen in electrical properties between the SPS-ed sample and the annealed one, suggesting that the samples prepared by MA and SPS in this work are not far the equilibrium state and their properties are stable for the analysis.

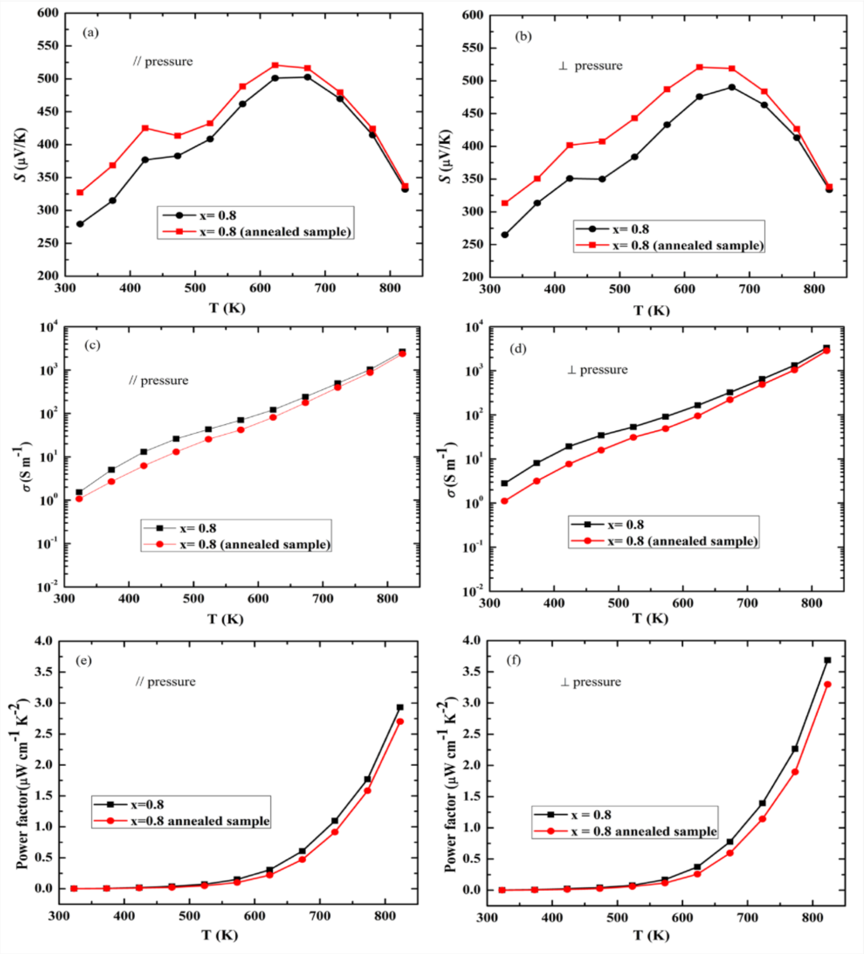

Figure S2 Temperature dependence of electrical properties of the two $SnS_{0.2}Se_{0.8}$ samples just after SPS (black) and subjected to annealing treatment (red) along (//, a, c, e) and perpendicular (⊥, b, d, f) to the SPS-pressurizing direction: (a, b) Seebeck coefficient, (c, d) electrical conductivity, (e, f) power factor.

References:

1 Zhao, L.-D. *et al.* Ultralow thermal conductivity and high thermoelectric figure of merit in SnSe crystals. *Nature* **508**, 373-377 (2014).

2 Orr, R. L. & Christensen, A. High Temperature Heat Contenits of Stannous and Stannic Sulfides. *J. Phys.Chem.* **62**, 124-125 (1958).

3 Lotgering, F. Topotactical reactions with ferrimagnetic oxides having hexagonal crystal structures—I. *J. Inorg. Nucl.Chem.* **9**, 113-123 (1959).
